# A Study on Cough Sensitivity and Airway Inflammation in Patients with Sinobronchial Syndrome

**DOI:** 10.1155/2022/2726261

**Published:** 2022-10-13

**Authors:** Shengyuan Wang, Shanshan Niu, Siwan Wen, Mengru Zhang, Cuiqin Shi, Zhongmin Qiu, Li Yu, Xianghuai Xu

**Affiliations:** Department of Pulmonary and Critical Care Medicine, Tongji Hospital, School of Medicine, Tongji University, Shanghai 200092, China

## Abstract

**Objective:**

This study aimed to clarify the characteristics of cough-reflex sensitivity and airway inflammation in patients with sinobronchial syndrome (SBS).

**Methods:**

39 patients with SBS, 53 patients with upper airway cough syndrome (UACS) induced by rhinitis, 33 patients with chronic sinusitis without cough, and 39 healthy controls (HCs) were enrolled between January 2013 and December 2018. All participants underwent a capsaicin cough-sensitivity test and cytology of induced sputum. The concentration of calcitonin-gene-related peptide (CGPR), histamine, prostaglandin (PG) E2, and eosinophil cationic protein (ECP) in induced sputum were measured using enzyme-linked immunosorbent assays (ELISAs).

**Results:**

The lowest concentration of capsaicin solution that induced ≥5 coughs (*C*5) was decreased markedly in patients with UACS induced by rhinitis compared with SBS patients (1.95 ± 2.92 *vs*. 31.2 ± 58.6 mol/L, *P* < 0.001), indicating higher cough-reflex sensitivity among UACS patients induced by rhinitis. However, there was no difference of these threshold between SBS patients and patients with sinusitis without cough and HCs. The percentage of neutrophils in sputum was increased remarkably in patients with SBS compared with HCs (40.0 ± 48.5% *vs*. 5.5 ± 9.0%, *P* < 0.001). A higher concentration of CGPR, histamine, and PGE2 was observed in induced sputum from patients with UACS induced by rhinitis than that in controls, and the ECP level was increased significantly in UACS induced by rhinitis compared with that in the other three groups.

**Conclusions:**

Cough-reflex sensitivity and airway inflammation in patients with SBS were different in patients with UACS induced by rhinitis. Thus, the mechanism of cough in those two patient populations might differ. Our study is registered in the Chinese Clinical Trials Register (https://www.chictr.org.cn/) as ChiCTR-TRC-00000152.

## 1. Introduction

“Sinobronchial syndrome” (SBS) was reported firstly by Japanese scholars in 1900 and named officially in 1966 [1]. SBS is characterized by chronic paranasal sinusitis combined with chronic nonspecific inflammation of the lower respiratory tract (LRT). The latter includes three types: chronic bronchitis, bronchiectasis, and diffuse panbronchiolitis [2]. Kartagener's syndrome, primary ciliary dyskinesia, Young's syndrome, and bronchoalveolar lavage lymphocytosis are all special types of SBS and are related to genetic factors [3]. Chronic bronchitis in the LRT is not characterized by obvious imaging abnormalities and is a common cause of chronic cough in Japan [4].

A unified consensus on SBS is not available [5]. A specific diagnosis of SBS has not been listed in cough guidelines from countries other than Japan. In most countries, cough caused by chronic sinusitis is believed to be similar to chronic cough caused by rhinitis and belongs to upper airway cough syndrome (UACS) [6–10]. Japanese guidelines recommend long-term and low-dose use of 14/15-membered ring macrolides to treat SBS [1]. For UACS caused by other nasal diseases, most national guidelines suggest treatment with histamine type-1 (H_1_) receptor antagonists [6–9]. UACS caused by sinusitis and UACS caused by rhinitis are treated differently, so we speculate that the mechanisms may differ, but few relevant studies have been conducted.

We wished to clarify the different characteristics between SBS and UACS caused by rhinitis, sinusitis without cough, and a healthy population.

## 2. Methods

### 2.1. Inclusion Criteria

All study participants met the following criteria: (i) aged 18–80 years; (ii) no wheezing, hemoptysis, or fever; (iii) no infections of the upper respiratory tract within 2 months of study commencement; (iv) no history of smoking or exposure to occupational dust or toxic/harmful gases; (v) no treatment with angiotensin-converting enzyme inhibitors in the previous 3 months; (vi) nonsmokers or individuals who quit smoking >2 years ago; (vii) no obvious abnormality in plain radiography or computed tomography (CT) of the chest; (viii) examination of pulmonary function: forced expiratory volume in one second (FEV_1_) >80% of the predicted value, FEV_1_/forced vital capacity >70%, and normal airway reactivity; (ix) no allergy to H_1_-receptor antagonists and no use of long-acting systemic or nasal H_1_-receptor antagonists or glucocorticoids in the previous 3 months; (x) no history of chronic obstructive pulmonary disease, bronchial asthma, interstitial pneumonia, or tuberculosis; (xi) nonpregnant and nonlactating women; (xii) no history of mental disorders.

### 2.2. Grouping of Patients

Patients were placed in one of the four groups.

#### 2.2.1. SBS and UACS Caused by Rhinitis

SBS patients (*n* = 39) and UACS patients (*n* = 53) caused by rhinitis at the Department of Respiratory and Critical Care Medicine of Tongji Hospital from January 2013 to December 2018.

The diagnostic criteria for UACS caused by SBS and UACS caused by rhinitis were according to Chinese Guidelines for the Diagnosis and Treatment of Cough (2009 and 2015 editions) and the diagnostic criteria of Japan [7, 11, 12]. Eight diagnostic criteria were used: (i) cough course >8 weeks with normal CT images; (ii) history of chronic rhinitis or sinusitis; (iii) one or more specific symptoms (sensation of secretions dripping down into the throat, throat-clearing sign, nasal discharge, nasal obstruction or sneezing); (iv) one or more specific signs (erythema and “cobblestone” appearance of the posterior pharyngeal mucosa, or mucoid/purulent secretions dripping into the pharynx); (v) mucosal blur/thickening >6 mm, or an air-fluid level on a radiograph of the sinuses; (vi) oral administration of H_1_-receptor antagonists (cetirizine, 10 mg, once-daily) with cough disappearance; (vii) chronic sinusitis that was treated efficaciously with macrolide drugs (erythromycin, 250 mg, b.d.); (viii) other causes of chronic cough were excluded.

**Figure 1:**
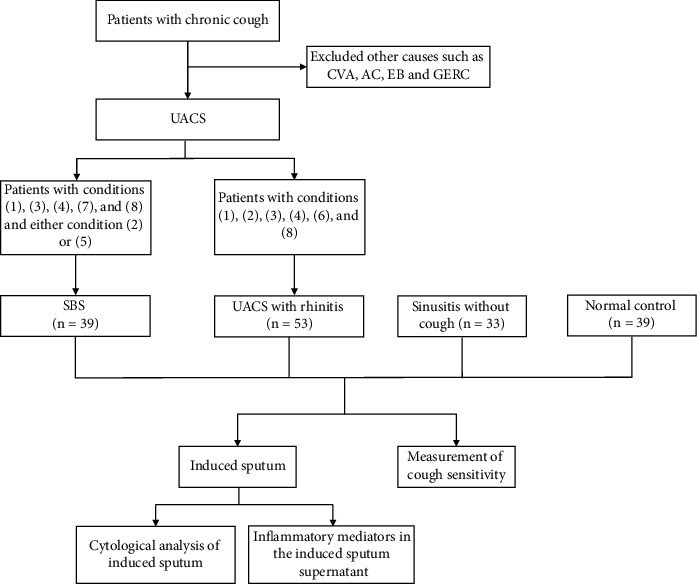
Consort diagram of the patients. (1) Cough course >8 weeks with normal CT images; (2) history of chronic rhinitis or sinusitis; (3) one or more specific symptoms (sensation of secretions dripping down into the throat, throat-clearing sign, nasal discharge, nasal obstruction, or sneezing); (4) one or more specific signs (erythema and “cobblestone” appearance of the posterior pharyngeal mucosa, or mucoid/purulent secretions dripping into the pharynx); (5) mucosal blur/thickening >6 mm, or an air-fluid level on a radiograph of the sinuses; (6) oral administration of H1-receptor antagonists (cetirizine, 10 mg, once-daily) with cough disappearance; (7) chronic sinusitis that was treated efficaciously with macrolide drugs (erythromycin, 250 mg, b.d.); (8) other causes of chronic cough were excluded.

Patients with conditions (i), (iii), (iv), (vii), and (viii) and either condition (ii) or (v) were diagnosed with SBS. Patients with conditions (i), (ii), (iii), (iv), (vi), or (viii) were diagnosed with UACS caused by rhinitis. The consort diagram of patients is presented as Figure 1.

#### 2.2.2. Sinusitis without Cough

From January 2013 to December 2018, 33 patients were diagnosed with chronic sinusitis at the Department of Otolaryngology in Tongji Hospital. The diagnosis was made by the chief otolaryngology physician based on the *Guidelines for the Diagnosis and Treatment of Chronic Rhinosinusitis* [13].

#### 2.2.3. Healthy Controls (HCs)

Thirty-nine healthy volunteers (hospital staff and medical students) were recruited from January 2013 to December 2018. None of these HCs had a history of allergy.

### 2.3. Measurements

#### 2.3.1. Measurement of Cough Sensitivity

A modified method proposed by our department, which was based on the method described by Fujimura et al. [14], was used. The participant was seated and wearing a nose-clip. Under calm breathing, an air compressor (PARI BOY®; Pari, Starnberg, Germany) at a compressor flow rate of 0.5 mL/min and particle diameter of 2 *μ*m was used for inhalation of atomized saline(0.9%) (base control). After confirmation of absence of cough, the participant inhaled a capsaicin solution (0.49 mmol/L) and the concentration doubled with each inhalation. The lowest concentration of capsaicin solution that induced ≥5 coughs was used as the cough threshold (*C*5), an indicator of cough sensitivity. A smaller value of C5 indicated a higher sensitivity to cough.

#### 2.3.2. Pulmonary Function and Bronchial-Provocation Test

Pulmonary function and bronchial-provocation tests using histamine were undertaken according to the guidelines developed by the American Thoracic Society [15] and the Chinese Thoracic Society [11]. The instrument used for this test was the MasterScreen™ pulmonary function testing system and the Aerosol Provocation System nebulizer from Jaeger (Berlin, Germany). A cumulative dose of histamine with a 20% decrease in FEV_1_ (PD20—FEV_1_) <7.8 *μ*mol was used as the standard to determine the increase in airway reactivity.

#### 2.3.3. Induction and Processing of Sputum

A modified method proposed by our department, which was based on the method described by Iredale et al. [16], was employed. The participant inhaled 4% hypertonic saline for 20–30 min *via* ultrasonic atomization. The nasal cavity was cleaned and the mouth rinsed every 5 min. The sputum expelled from the lungs by coughing was collected in sterile, clean containers. Then, 0.1% dithiothreitol (which is a Bowman–Birk protease inhibitor) was added to sputum samples at 4 × the volume of sputum. Samples were mixed by pipetting, placed in a constant-temperature water-bath at 37°C for 15 min, and swirled and shaken for 15 min. Phosphate-buffered saline (PBS; 2 × volume) was added, and samples were mixed and incubated in a constant-temperature water-bath at 37°C for 5 min. Samples were filtered through a 48 *μ*m nylon mesh and centrifuged at 1500 rpm for 10 min at room temperature. The supernatant was stored at −80°C until further testing.

#### 2.3.4. Cytology of Induced Sputum

The total number of cells in induced sputum was calculated using a hemocytometer. Cells were suspended in PBS, and cell viability was examined using trypan blue staining. The cell suspension was placed on glass slides in a cell centrifuge (Shandon™ II; Thermo Scientific, Waltham, MA, USA). After drying in air, cells were fixed with 4% paraformaldehyde and stained with hematoxylin and eosin (H&E). Under a microscope, 400 nucleated cells were classified and counted. The percentages of macrophages, neutrophils, eosinophils, and lymphocytes were calculated.

#### 2.3.5. Detection of Inflammatory Mediators in the Supernatant of Induced Sputum

The supernatant of induced sputum was thawed at room temperature. Enzyme-linked immunosorbent assays (ELISAs) were conducted strictly according to the manufacturer's (Shanghai Yuan-Yang Medical Devices, Shanghai, China) instructions. The intrabatch error was <5%, and the interbatch error was <10%, for all ELISA kits. The sensitivity of each protein was 7.8 pg/mL for calcitonin gene-related peptide (CGRP), 0.01 ng/mL for histamine, 0.01 pg/mL for prostaglandin (PG) E2, 7.8 pg/mL for interleukin (IL)-8, and 1.5 ng/mL for eosinophil cationic protein (ECP).

### 2.4. Procedure

General clinical assessments (medical history, chest imaging, pulmonary-function tests, and bronchial-provocation test) were completed. Subsequent steps were established according to the diagnosis and treatment of chronic cough and rhinitis-sinusitis, including SBS; UACS caused by rhinitis; cough-sensitivity tests; examination of induced sputum after the initial diagnosis of sinusitis; cytology of induced sputum; detection of inflammatory mediators in induced-sputum supernatant. The corresponding treatment was provided according to different etiologies. Weekly follow-up was conducted at the outpatient clinic. The diagnosis was established for patients who exhibited a strong response to the treatment.

### 2.5. Statistical Analyses

The calculation of the sample size was based on the data in our published observation [17] and the literature [18]. Age and lung-function indicators are presented as the mean ± SD. The cough course is presented as the median (25–75% interquartile). *C*2 and *C*5 were log-transformed to normalize the data and presented as geometric mean ± SD. After testing, the cytological classification of induced sputum and levels of inflammatory mediators in the supernatant had a nonnormal distribution and are reported as the median (25–75% interquartile). The *t*-test was used to compare data between the two groups when variances were uniform, and the Mann–Whitney *U* test was used to compare data between the two groups when variances were uneven. Multiple groups were compared by using the Kruskal–Wallis H test. Statistical analyses were undertaken with SPSS 20.0 (IBM, Armonk, NY, USA). *P* < 0.05 was considered significant.

## 3. Results

### 3.1. Demographic Details

The general information for each group is provided in Table 1. Significant differences in age, gender, cough course, LCQ score, cough symptom score, and pulmonary function were not observed between the groups.

### 3.2. Comparison of Cough Sensitivity among Different Groups

**Figure 2:**
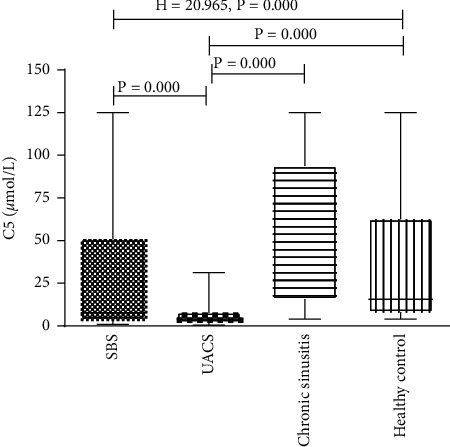
Cough sensitivity for each group.

Significant differences (*H* = 20.965, *P* < 0.001) in *C*5 were observed among the different groups. *C*5 for the groups SBS (11.671 ± 0.445), sinusitis without cough (41.210 ± 0.283), and normal control (19.679 ± 0.301) were significantly higher than the value for the UACS-caused-by-rhinitis group (3.428 ± 0.294) (*P* < 0.001 for all) (Figure 2).

### 3.3. Comparison of the Classification of Cells in Induced Sputum from Each Group

Significant differences in the total numbers of cells were observed between each group (*H* = 82.715, *P* < 0.001). The percentage of neutrophils among the different groups was also significantly different (*H* = 38.726, *P* < 0.001). Significantly higher percentages of neutrophils were observed in the groups of SBS (*P* < 0.001) and sinusitis without cough (*P*=0.022) than those in the normal-control group. In addition, a significantly higher percentage of neutrophils was observed in the SBS group than that in the UACS-caused-by-rhinitis group (*P* < 0.001). The percentage of eosinophils was significantly different among different groups (*H* = 15.445, *P*=0.001) (Table 2).

### 3.4. Comparison of Levels of Inflammatory Mediators in the Induced-Sputum Supernatant from Each Group

In the induced-sputum supernatants from various groups, the concentration of histamine (*H* = 29.694, *P* < 0.001), PGE2 (*H* = 14.244, *P*=0.003), and CGRP (*H* = 9.182, *P*=0.014) was significantly different. The ECP concentration was also significantly different (*H* = 32.809, *P* < 0.001) in the induced-sputum supernatants from various groups (Table 3).

## 4. Discussion

The characteristics of patients in the groups of SBS and UACS caused by rhinitis differed, even though both conditions are included in the same syndrome (UACS) in most guidelines from different countries. In the SBS group, the cough sensitivity did not increase significantly, and the percentage of neutrophils in induced sputum was high. The cough sensitivity of patients with UACS caused by rhinitis was significantly higher than that of participants in the normal-control group, and the percentage of eosinophils in induced sputum as well as levels of CGRP, histamine, and PGE2 in the supernatant were higher than that in the normal-control group. These findings are consistent with the results from our previous study, which showed that UACS caused by rhinitis may be related to activation of the mast cells [19].

Several studies have concluded that the cough sensitivity of patients with UACS increases significantly compared with patients with SBS [5, 17]. Similarly, our study validated that the cough sensitivity of patients with UACS caused by rhinitis increased, whereas the cough sensitivity of patients with SBS did not increase significantly. The capsaicin used as the cough-inducing agent in our study mainly stimulates transient receptor potential vanilloid-1 on afferent vagal C fibers to produce the cough reflex. However, capsaicin does not affect another sensor on C fibers (transient receptor potential ankyrin subtype 1 protein) or A*δ*-fibers (which are more sensitive to mechanical stimulation in the airways). As mentioned above, the airways of patients with SBS presented a hypersecretory phenotype, which may induce coughing by stimulating A*δ*-fibers. The probability of paranasal sinus secretions entering the LRT is very small [20]. However, compared with other causes of chronic cough, mucin synthesis and secretion occur mainly in the LRT of patients with SBS [21]. Animal experiments have shown that preventing secretions from entering the lower airways reduces the number of coughs significantly [22]. Therefore, patients with SBS may develop a cough due to stimulation of A*δ*-fibers. In addition, due to many secretions in the airways, inhaled capsaicin aerosol may not completely contact submucosal transient receptor potential vanilloid-1, thereby affecting the results of cough-sensitivity detection.

Consistent with the high percentage of neutrophils in induced sputum for the SBS group, relative studies have showed that neutrophilic inflammation was present in the lower airways of patients with SBS; whereas the increase in the number of neutrophils in the airway is not necessarily attributed to respiratory infections as patients in the SBS group did not have infection-related manifestations, such as fever and yellow sputum, and received with standard antiinfection regimens. Coughing itself can produce mechanical damage to the airway mucosa [23]. Studies using cough models in guinea pigs (including studies undertaken in our department) also showed that coughing causes airway damage, produces neutrophilic airway inflammation, and improves the hypersensitivity of cough reflexes [24]. However, patients with sinusitis without cough also presented an increased proportion of lower-airway neutrophils, and patients with UACS caused by rhinitis had cough but a low proportion of neutrophils. Therefore, mechanical injury to the airways does not completely explain the increase in the number of neutrophils. Further mechanism could be explained as a low level of bacterial colonization in patients with chronic sinusitis, resulting in the secretion of IL-8, local recruitment of neutrophils, and macrophage dysfunction. Besides, neutrophil metabolism is related to the expression of receptor for advanced glycation end products (RAGE) [25]. More studies are needed to ascertain if the upregulation of RAGE expression in the lower airways of patients with SBS results in increased percentages of neutrophils.

The mechanism of SBS-induced cough is not clear. Previously, nasopharyngeal secretions were postulated to stimulate the throat or enter the lower airways, thereby causing cough [11]. Hara et al. observed >100 patients with sinusitis, and most patients had postnasal drip but did not cough. According to Bardin et al., the secretions of patients with sinusitis rarely enter the lower airways and often enter the digestive tract [20]. Therefore, some scholars have proposed that lower airway inflammation may be a possible mechanism of cough [17].

Based on the results of *in vivo* and *in vitro* experiments, macrolides improve macrophage phagocytosis and downregulate expression of intercellular adhesion molecule-1 and IL-8, eventually inducing neutrophil apoptosis [26] and alleviating neutrophilic airway inflammation in individuals [27]. In the present study, cough resolved after patients with SBS were treated with macrolides. Therefore, we speculated that macrolides relieved coughing by inhibiting neutrophilic inflammation in the airways. However, we did not retest induced sputum after the cough had resolved, which should be conducted in future studies.

Although the proportion of neutrophils in the airways of patients with SBS increased, the cough sensitivity measured by the capsaicin test did not increase, suggesting that neutrophils might cause coughing through other methods. Yousaf et al. [27] failed to alleviate the symptoms of idiopathic cough although low-dose macrolide drugs inhibited neutrophilic inflammation, suggesting that neutrophils may not be associated with coughing. Neutrophils may be the cause of coughing or may only accompany wth coughing.

Our study had three main limitations. Firstly, we did not retest the induced sputum from patients with SBS after treatment and did not further confirm the causal relationship between neutrophilic airway inflammation and cough. Secondly, immunoglobulin-E detection was not done in any serum samples. Therefore, the effect of allergic factors on cough sensitivity in each group requires further study.

## 5. Conclusions

The cough sensitivity of patients with SBS was not high. Cough symptoms may be generated by some unknown pathways, which require further in-depth study. High-quality randomized controlled trials demonstrating the efficacy of low-dose macrolides for SBS are lacking. Nevertheless, our study at least describes the differences in characteristics between patients with SBS and rhinitis-induced UACS, indicating possible differences in the treatment between these conditions and providing a theoretical basis for future studies.

## Figures and Tables

**Table 1:** General information for each group.

Indicators	SBS group	UACS rhinitis group	Sinusitis group	Normal control group
Case number (male)	39 (18)	53 (18)	33 (13)	39 (13)
Age (years)	50.4 ± 12.6	45.2 ± 14.3	47.9 ± 16.4	40.2 ± 15.8
Cough course (months)	10 (90)	6 (46)	—	—
LCQ score	65.70 ± 13.36	68.38 ± 16.43	—	—
Cough symptom score				
Daytime	3.00 (1.00)	3.00 (1.25)	—	—
Nighttime	1.00 (1.25)	1.00 (1.00)	—	—
FEV1% predicted	86.9 ± 14.9	98.4 ± 14.3	95.2 ± 16.0	94.8 ± 11.3
FVC% predicted	91.5 ± 14.8	103.9 ± 18.1	100.0 ± 11.1	95.8 ± 11.3
FEV1/FVC%	80.8 ± 13.2	80.5 ± 8.1	77.2 ± 10.2	84.1 ± 6.0
BMI (kg/m^2^)	20.9 ± 1.7	22.1 ± 2.3	21.3 ± 1.9	21.8 ± 1.5

Note: cough course is presented as the median (25–75% interquartile), and the other data are expressed as the mean ± standard deviation—no data shown.

**Table 2:** Classification of induced sputum cells in each group.

Indicators	SBS group	UACS rhinitis group	Sinusitis group	Normal control group	*P* values
Total cell number(^*∗*^10^6^/L)	3.9 (3.6)^*∗*^	4.1 (3.8)^*∗*^	5.5 (9.7)^*∗*^	2.1 (1.3)	<0.001
N (%)	40.5 (48.5)^*∗*^^#^	5.5 (10.5)	27.5 (51.0)^*∗*^	5.5 (9.0)	0.002
M (%)	21.5 (45.0)^*∗*^^#^	65.0 (35.0)^*∗*^	45.0 (29.0)^*∗*^	77.5 (15.0)	<0.001
L (%)	17.5 (27.0)	19.0 (12.5)	13.5 (17.0)	17.0 (16.5)	0.670
E (%)	0 (0.5)	0.5 (2.5)^*∗*^	0 (0.5)	0 (0)	0.003

Note: all the data are presented as the median (25–75% interquartile). ^*∗*^ compared with the normal-control group, *P* < 0.05, ^#^ compared to the UACS rhinitis group, *P* < 0.05; N: neutrophils, M: monocytes, L: lymphocytes, and E: eosinophils.

**Table 3:** Comparison of inflammatory mediators in the induced sputum supernatants in each group.

Indicators	SBS group	UACS rhinitis group	Sinusitis group	Normal control group
ECP (pg/ml)	1426.0 (1090.0)^#^	2492.0 (809.0)	1429.0 (977.0)^#^	1040.0 (1274.8)^#^
IL-8 (pg/ml)	52.5 (29.0)	25.2 (46.9)	48.1 (36.6)	47.3 (36.2)
CGRP (pg/ml)	28.8 (10.8)	37.0 (21.8)^*∗*^	29.3 (7.3)	25.6 (9.5)
Histamine (ng/ml)	873.0 (1197.0)	1125.0 (901.0)^*∗*^	372.0 (295.0)	271.0 (300.0)
PGE2 (pg/ml)	84.6 (71.5)	83.5 (60.2)^*∗*^	67.8 (79.8)	38.5 (35.1)

Note: all the data are presented as the median (25–75% interquartile). ^*∗*^ compared with the normal-control group, *P* < 0.05, ^#^ compared with the UACS rhinitis group, *P* < 0.05. PGE2: prostaglandin E2, ECP: eosinophil cationic protein, CGRP: calcitonin gene-related peptide, and IL-8: interleukin 8.

## Data Availability

The data that support the findings of this study are available from the corresponding author upon reasonable request.
